# Vulnerability of Homeless People in Tehran, Iran, to HIV, Tuberculosis and Viral Hepatitis

**DOI:** 10.1371/journal.pone.0098742

**Published:** 2014-06-04

**Authors:** Fahimeh Bagheri Amiri, Mohammad Mehdi Gouya, Mahnaz Saifi, Mehdi Rohani, Payam Tabarsi, Abbas Sedaghat, Noushin Fahimfar, Arash Memarnejadian, Mohammad Reza Aghasadeghi, Ali Akbar Haghdoost, Fatemeh Jahanbakhsh, Mahshid Nasehi, Ehsan Mostafavi

**Affiliations:** 1 Department of Epidemiology, Pasteur Institute of Iran, Tehran, Iran; 2 Department of Epidemiology, University of Tehran, Tehran, Iran; 3 Center for Disease Control, Ministry of Health and Medical Education, Tehran, Iran; 4 Department of Tuberculosis and Lung Research, Pasteur Institute of Iran, Tehran, Iran; 5 Department of Bacteriology, Pasteur Institute of Iran, Tehran, Iran; 6 Mycobacteriology Research Center, National Research Institute of Tuberculosis and Lung Disease, Shahid Beheshti University of Medical Science, Tehran, Iran; 7 Department of Hepatitis & AIDS, Pasteur institute of Iran, Tehran, Iran; 8 Regional Knowledge Hub, and WHO Collaborating Centre for HIV Surveillance, Institute for Futures Studies in Health, Kerman University of Medical Sciences, Kerman, Iran; 9 Research Center for Modeling in Health, Institute for Futures Studies in Health, Kerman University of Medical Sciences, Kerman, Iran; 10 Department of Epidemiology and Biostatistics, School of public Health, Tehran University of Medical Sciences, Tehran, Iran; 11 Research Centre for Emerging and Reemerging Infectious Diseases (Akanlu), Pasteur Institute of Iran, Kabudar Ahang, Hamadan, Iran; University of California, San Francisco, United States of America

## Abstract

**Background:**

Homeless people are at risk of contracting communicable infectious diseases, as they indulge in risky behaviours and lifestyle. This study was conducted to determine the prevalence of the aforementioned infections and related risk behaviours among homeless people in Tehran.

**Methods:**

In this study a convenience sample of 593 homeless individuals was studied. The ELISA method was used for the detection of HIV, HCV and HBV. Clinical symptoms, sputum cultures, acid fast bacilli smears, and chest X-rays were used to identify active pulmonary tuberculosis, and the Interferon Gamma Release Assay (IGRA) test was used to identify latent tuberculosis.

**Results:**

The prevalence of HIV, HBV, HCV and latent tuberculosis was 3.4%, 2.6%, 23.3% and 46.7%, respectively. Active pulmonary tuberculosis was found in 7 persons (1.2%). Injection drug use was an independent risk factor for HIV, HCV and HBV infections. Older people had a higher proportion of Mycobacterium tuberculosis infection (OR: 2.6, 95%CI: 1.9, 3.7) and HCV positivity (OR: 1.7, 95% CI: 1.1, 2.5).

**Conclusion:**

Our findings highlighted that much more attention needs to be paid to the health of homeless people.

## Introduction

Homeless people are individuals who either have no place to sleep, or sleep in public or private shelters [1]. An estimated 100 million people are homeless worldwide [2]. The fatality rate among this group of people is almost 4 times higher than the general population [3].

Intravenous drug use and risky sexual behaviour increases the risk of infection with blood-borne infectious diseases such as HIV and viral hepatitis B and C [4], [5]. In addition, low levels of education, poor housing conditions, low income, poor hygiene, inappropriate employment, poor nutrition and limited access to health services increase vulnerability to infections such as TB and HIV [6]. Homeless people are at risk of these communicable infectious diseases, as they have the aforementioned risky behaviours and life style. Therefore, appropriate health interventions should be applied for homeless people to reduce the adverse outcomes that result from these infections [2], [3], [7]. TB infection among the homeless can lead to a wider public health problem [8], [9].

Self-esteem, family attachment, weak social networks, drug abuse, and childhood criminal victimisation were all significantly related to homelessness in Iran [10]. Usually homeless people will be homeless for ever. There is no estimate of the number of homeless people and the rate of their risky behaviours in Iran. Based on our best knowledge, the only biological survey on homeless people in Tehran was a study of HIV, HBV, HCV, syphilis, and associated risky behaviours on 202 homeless men conducted in 2007 [7]. Due to a lack of updated information on blood-borne viral infections, and the absence of a study on the situation of TB infection in homeless people in Iran, the aim of this study was to assess the prevalence of HIV, latent and active tuberculosis, hepatitis B, and hepatitis C, as the most important infections among this group, and to evaluate the high-risk behaviours associated with these infections in homeless people in Tehran.

## Materials and Methods

### Study area

This study was conducted in Tehran. Tehran is the capital of Iran and is located in the north of the country. The population of this city was estimated to be approximately9 million in 2011.

### Study Population

Participants were recruited from five centres working with the authority of the municipality of Tehran from June to August 2012. Homeless people were eligible to participate in the study if they were 18–60 years old and had been continuously or discontinuously homeless (during the month prior tothe study period) for at least 10 days. In this study, as most of the participants were either illiterate or had a low level of educational, verbal informed consent was provided. This form included descriptions of voluntary participation, incentives for participating at any stage, and the possibility of free treatment of TB patients and HIV infected persons. A refusal to participate in the study did not exclude anvone from treatment. In this study, a homeless person was defined as someone who had no home or shelter to reside in, and instead resided on the corners of streets, in parks, or in public places, if there was no designated residence provided by governmental or non-governmental organisations.

### Data Collection

A researcher-made questionnaire was used to assess behaviours and risk factors. Questions included sociodemographic characteristics, putative risk factors for the studied infection (history of substance abuse, criminal records, sexual relations and high-risk behaviours related to these variables), and the presence of TB-related symptoms. The questionnaire was in Persian and carried out face to face in a quiet place.

The ethical committee of Pasteur Institute of Iran approved the consent procedure and the proposal and protocol of the study, covering all the samples taken (blood, sputum, x-rays, etcs), in addition to the questionnaire.

Informed consent was given by the participants to use their clinical records. Incentives were paid to participants at each stage of the study.

Blood samples were taken to determine the prevalence of HIV, HBV, HCV and latent TB infection. Sputum samples were collected twice from individuals wo had either the main clinical signs of tuberculosis (fever, cough persisting for more than two weeks, night sweats and weight loss) or a positive result for the HIV rapid test. All participants were transported to a nearby radiological centre for chest radiography. A portable X-ray was used in cases where it was not possible to send participants to a radiography centre. The diagnosis of pulmonary TB was performed by an infectious disease specialist, based on clinical signs, chest X-ray result, acid-fast staining, culture results (after two months) and Quantiferon results. Individuals with clinical and radiographic signs indicative of tuberculosis, but no positive cultures or smears were presented as suspected tuberculosis patients.

### Laboratory Testing

Sure check HIV-1/2 (Chembio Diagnostics Systems, New York, USA) was used as the first test for HIV in sampling locations. HIV positive results were confirmed with ELISA test (Genscreen Ultra HIV Ag-Ab, BioRad, France), which detects both antigen and antibody HIV1, 2. Blood samples were double tested to detect hepatitis C virus antibody and hepatitis B surface antigen (HBsAg) using commercially available ELISA kits (DIA PRO Diagnostic Bioprobes, Srl., Italy). *Mycobacterium tuberculosis* infection was detected using the IGRA test (QuantiFERON TB Gold In-Tube [QFT] assay; Cellestis Ltd., Carnegie, Victoria, Australia) for all participants. Sputum AFB smears and cultures were carried out for suspected cases. In cases of positive culture results for *Mycobacterium tuberculosi*s, an antibiogram was carried out using six antibiotics [Isoniazid (INH); Rifampin (RMP); Streptomycin (SM); Ethambutol (EMB); Ethionamide (Eth); Kanamycin (KM)] by a proportional method.

### Statistical Analysis

Data analysis was carried out by SPSS (version 16, SPSS Inc, Chicago) software package. Associations between participants' characteristics and the infections were evaluated using logistic regression and chi-square test. P-values less than 0.05 were considered statistically significant. Variables with p-values less than 0.10 in univariate models were considered for multivariate analysis. A backward stepwise model was used to detect the main effects in the final model.

## Results

### Descriptive results

In this study, 593 homeless people (513 males and 80 females, all Iranian nationals) were enrolled from Tehran. Median age and length of time of homelessness was 41 years and 24 months, respectively. 64% of participants had an education level lower than high school and 11.8% were illiterate. 36.1% were using drugs at the time of study. At last drug use, the most frequent type of drug used was methamphetamine (45.5%). 27.5% of individuals with a lifetime history of drug use had a history of injecting drugs, and over half of those (52.5%) had a history of sharing needles. Median age at first injection was 29 years. 85.5% of participants were smoking at the time of study. 48.8% of participantshad a history of incarceration, the median duration of which in the 10 years prior to the period of study was 11 months. 79.8% of individuals had a history of sexual contact; of these, 14 women (21.9%) and 36 men (9.3%) had a history of either selling sex or having sex with other men, respectively. Out of all participants, 60.37% had been married and 21.5% had a history of sex outside marriage.

### Prevalence of HIV, HBV, HCV and TB

The prevalence of HIV, HBV, HCV, IGRA positivity and active pulmonary TB were 3.4% (95% CI: 2.1%, 5.1%), 2.58% (95% CI: 1.5%, 4.1%), 23.3% (95% CI: 20.0%, 26.9%), 46.7% (95% CI: 42.3%, 50.4%) and 1.2% (95% CI: 0.5%, 2.3%), respectively. Five participants (0.8%) were diagnosed as suspected cases for active tuberculosis, but due to lack of access to them for further examination, as they were no longer available in the sampling centres, follow up of their cases for confirmation was not possible. Four persons (0.7%) were culture positive for *M. tuberculosis*, two of whom were resistant for SM only. Five individuals (0.8%) had at least one positive smear for acid-fast staining.

### Prevalence of co-infections with HIV

A total of 17 (2.9%, 95% CI: 1.8, 4.5), 3 (0.5%, 95% CI: 0.1%, 1.4%), 3 (0.5%, 95% CI: 0.1%, 1.4%) and 7 (1.2%, 95% CI: 0.5%, 2.4%) persons were co-infected with HIV/HCV, HIV/HBV, HIV/HBV/HCV and HIV/IGRA+, respectively. Only one participant was co-infected with HIV and active TB.

### HIV risk factors

Univariate analysis showed that the use of Kerack (a derivative of heroin) and injecting drug use increased the risk of HIV positivity 9.1 (OR: 9.1, 95% CI: 1.8, 46.3) and 8.2 (OR: 8.2, 95% CI: 2.9, 23.4) times, respectively. Although HIV prevalence in females who sold sex was over 3 times higher than in other women and its prevalence in men who have sex with men (MSM) was more than twice higher than in other men, these differences were not statistically significant. Significant differences were not found between HIV positivity and other behavioural variables (**table 1**). A positive association was seen between uthe use of Kerack and a history of injecting drug use (OR: 2.5, 95% CI: 1.2, 5.0).

**Table 1:** Univariate analysis of factors associated with the prevalence of HIV, HCV, HBV and latent TB on homeless people in Tehran.

Variables	Categories	HIV	OR (95% CI)	Latent TB	OR (95% CI)	HBV	OR (95% CI)	HCV	OR (95% CI)
Age*	**More than 41 year**	7(2.45)	0.54(0.21,1.38)	164(58.36)	2.62(1.87,3.68)	8(2.83)	1.18(0.42,3.30)	80(28.36)	1.69(1.14,2.50)
	**Less than 41 years**	13(4.41)		100(34.84)		7(2.41)		55(18.97)	
Gender	**Female**	2(2.50)	1.42(0.32,6.23)	11(13.75)	6.80(3.51,13.15)	1(1.25)	2.27(0.29,17.48)	10(12.50)	2.35(1.17,4.69)
	**Male**	18(3.51)		259(52.01)		14(2.79)		126(25.10)	
Educational level	**Illiterate**	2(2.94)	Reference	35(52.23)	Reference	3(4.47)	Reference	13(19.40)	Reference
	**Literate**	0(0.00)	-	13(44.82)	0.74(0.31.1.78)	1(3.44)	0.76(0.08,7.65)	4(13.79)	0.67(0.20,2.24)
	**Primary school**	5(4.31)	1.48(0.28,7.88)	50(43.85)	0.71(0.39,1.31)	2(1.73)	0.38(0.06,2.32)	25(21.74)	1.15(0.55,2.44)
	**Secondary school**	4(2.58)	0.87(0.16,4.89)	75(49.66)	0.90(0.51,1.61)	6(3.92)	0.87(0.21,3.59)	39(25.49)	1.42(0.70,2.88)
	**High school**	6(3.87)	1.33(0.26,6.76)	68(45.33)	0.76(0.43,1.35)	3(1.99)	0.43(0.09,2.20)	33(21.85)	1.16(0.57,2.38)
	**Academic level**	1(1.92)	0.65(0.06,7.34)	23(45.09)	0.75(0.36,1.56)	0(0.00)	-	18(35.29)	2.27(0.98,5.22)
Duration of being Homeless*	**More than 24 month**	11(4.38)	1.66(0.68,4.06)	140(56.56)	2.07(1.48,2.89)	7(2.83)	1.17(0.42,3.27)	66(26.72)	1.35(0.92,1.99)
	**Less than 24 month**	9(2.69)		126(38.77)		8(2.43)		70(21.28)	
**History of drug use**									
Drug use (history of)	**Yes**	18(3.97)	2.69(0.62,11.75)	65(50.00)	0.84(0.57,1.24)	11(2.46)	0.79(0.25,2.52)	116(25.95)	1.92(1.14,3.22)
	**No**	2(1.53)		203(45.59)		4(3.13)		20(15.63)	
Drug use (currently)	**Yes**	8(3.74)	0.88(0.34,2.26)	77(36.84)	0.51(0.35,0.75)	6(2.84)	1.35(0.41,4.50)	46(21.80)	0.65(0.43,1,02)
	**No**	10(4.18)		125(53.41)		5(2.11)		70(29.66)	
**Kind of drug used**									
Hashish	**Yes**	1(7.14)	2.12(0.24,18.56)	7(50.00)	1.79(0.6,5.30)	0(0.00)	2.67(0.10,20.41)	3(21.42)	0.98(0.26,3.66)
	**No**	7(3.50)		70(35.90)		6(3.04)		43(21.82)	
Kerack**	**Yes**	6(10.34)	9.06(1.77,46.26)	13(22.41)	0.39(0.20,0.78)	2(3.45)	1.35(0.24,7.47)	12(20.69)	0.91(0.44,1.92)
	**No**	2(1.28)		64(42.38)		4(2.61)		34(22.22)	
Heroin	**Yes**	4(5.26)	1.86(0.45,7.67)	30(40.54)	1.28(0.71,2.28)	2(2.70)	0.92(0.16,5.13)	14(18.91)	0.76(0.38,1.54)
	**No**	4(2.90)		47(34.81)		4(2.92)		32(23.36)	
Methamphetamine	**Yes**	4(4.12)	1.17(0.28,4.79)	27(27.83)	0.48(0.27,0.86)	5(5.15)	5.96(0.68,51.89)	17(17.52)	0.62(0.31,1.21)
	**No**	4(3.45)		50(45.04)		1(0.88)		29(25.66)	
Opium	**Yes**	2(2.40)	0.53(0.10,2.67)	28(35.00)	0.87(0.49,1.56)	2(2.44)	0.79(0.14,4.43)	20(24.39)	1.30(0.67,2.53)
	**No**	6(4.58)		49(37.98)		4(3.10)		26(20.15)	
Others	**Yes**	1(6.66)	1.99(0.23,11.33)	5(33.33)	0.84(0.28,2.56)	0(0.00)	2.4(0.10,18.24)	6(40.00)	2.63(0.89,7.83)
	**No**	7(3.52)		72(37.11)		6(3.06)		40(20.41)	
Injecting drugs	**Yes**	14(11.38)	8.22(2.89,23.35)	62(51.66)	1.39(0.92,2.13)	7(5.83)	3.93(1.22,12.62)	61(50.83)	4.25(2.71,6.68)
	**No**	5(1.54)		138(43.39)		5(1.55)		63(19.65)	
Sharing needle	**Yes**	9(14.06)	1.74(0.55,5.51)	33(52.38)	1.10(0.54,2.26)	5(7.94)	2.33(0.43,12.5)	34(53.97)	1.26(0.61,2.59)
	**No**	5(8.62)		28(50.00)		2(3.57)		27(48.21)	
**Incarceration**									
Incarceration history	**Never**	8(2.68)	Reference	118(40.41)	Reference	6(2.04)	Reference	49(16.72)	Reference
	**More than 10 years ago**	2(2.90)	1.08(0.23,5.21)	36(52.17)	1.61(0.95,2.73)	2(2.90)	1.43(0.28,7.23)	16(23.18)	1.50(0.79,2.84)
	**Recent 10 years**	10(4.65)	1.77(0.69,4.56)	111(53.36)	1.69(1.18,2.42)	7(3.32)	1.64(0.54,4.96)	28(26.41)	2.47(1.63,3.76)
Duration of Incarceration (in last 10 years) *	**Upper than 11 month**	6(5.71)	1.58(0.43,5.75)	65(63.10)	2.12(1.21,3.70)	2(1.94)	0.40(0.08,2.11)	70(33.17)	1.92(1.07,3.44)
	**Lower than 11 month**	4(3.70)		46(44.66)		5(4.72)		42(40.77)	
**Sex history**									
Having sex (history of)	**Yes**	14(3.00)	0.58(0.22,1.54)	226(49.67)	1.89(1.24,2.89)	12(2.61)	1.02(0.28,3.68)	28(23.93)	0.98(0.61,1.58)
	**No**	6(5.08)		40(34.18)		3(2.56)		108(23.52)	
Condom use at last sexual encounter	**Yes**	6(3.90)	1.53(0.52,4.49)	68(44.73)	0.74(0.50,1.10)	1(0.66)	0.18(0.02,1.38)	36(23.68)	1.02(0.64,1.61)
	**No**	8(2.58)		156(52.17)		11(3.63)		71(23.43)	
Sex work (Female)	**Yes**	1(7.14)	0.27(0.02,4.53)	1(7.14)	2.48(0.28,21.69)	0(0.00)	0.00(0.00,67.25)	2(14.28)	0.82(0.15,4.58)
	**No**	1(2.00)		8(16.00)		1(2.00)		6(12.00)	
Men who have sex with men	**Yes**	2(5.56)	0.49(0.04,5.83)	18(50.0)	1.27(0.64,2.52)	2(5.56)	0.46(0.09,2.2)	7(19.44)	1.42(0.60,3.37)
	**No**	9(2.56)		190(55.88)		9(2.61)		88(25.58)	

*Median of the variable is used for analysis.

**A purified and potent form of heroin (not to be mistaken with crack cocaine).

In multivariate analysis, the use of Kerack was still a risk factor for HIV positivity (OR: 7.0, 95% CI: 1.3, 37.2).

### HBV risk factors

Univariate analysis showed that injecting drug use increased the risk of HBV infection (OR: 3.9, 95% CI: 1.2, 12.6); however in multivariate analysis there were no factors related to HBV positivity.

### HCV risk factors

Older people had a higher rate of HCV positivity (OR: 1.7, 95% CI: 1.1, 2.5). HCV positivity was higher in men (OR: 2.4, 95% CI: 1.2, 4.7). A positive association was found between HCV positivity and a history of drug use (OR: 1.9, 95% CI: 1.1, 3.2), injecting drug use (OR: 4.3, 95% CI: 2.7, 6.7) and duration of incarceration in the past 10 years (OR: 1.9, 95% CI: 1.1, 3.4). In addition, individuals with a history of incarceration in the last 10 years had a HCV positivity rate 2.5 times higher than those with no history of incarceration.

In multivariate analysis, older people were still more at risk of HCV (OR: 2.8, 95% CI: 1.4, 5.2) and a history of injecting drug use, increased the risk of HCV positivity 4.0 times (OR: 4.0, 95% CI: 2.0, 8.1) (**table 1**).

### Latent TB risk factors

Univariate analysis showed a higher risk of IGRA+ in older persons (OR: 2.6, 95%CI: 1.9, 3.7) and in individuals who had a longer duration of homelessness (OR: 2.1, 95%CI: 1.5, 2.9). Men had a rate 6.8 times higher than women IGRA+ (OR: 6.8, 95% CI: 3.5, 13.2). Duration of incarceration had a positive association with being IGRA+ (OR: 2.1, 95% CI: 1.2, 3.7). Individuals with a history of incarceration in the last 10 years had a IGRA positive rate 1.7 times higher than those with no history of incarceration. People with a history of sexual contact had a higher rate of IGRA+ (OR: 1. 9, 95% CI: 1.2, 2.9) (**table 1**).

In multivariate analysis, individuals with a longer duration of incarceration had an 22.2 times higher IGRA+ (OR: 22.2, 95% CI: 3.3, 149.0) rate.

## Discussion

The results of this study showed that the prevalence of hepatitis C and latent tuberculosis amongst homeless people of Tehran was considerably high, with almost a quarter of participants having hepatitis C infection and almost half of them having latent tuberculosis. High-risk behaviours increased the risk of infection, and it was shown that drug injection was also a major risk factor for hepatitis B, hepatitis C and HIV in the study group.

The Municipality of Tehran has provided shelters for homeless people, which offer a bed, two meals a day, health services, and counselling. It should be noted that when people gather together in one place, if there is a TB patient among them, the disease can spread rapidly. About 50 percent of the participants of this study had a latent tuberculosis infection; these people were at risk of developing the clinical form of tuberculosis, because of poor nutrition, drug abuse, inadequate housing and other risk factors. Therefore, the detection and treatment of clinical tuberculosis patients and improvement of the surveillance system of infections such as tuberculosis and HIV in this group can improve the health of homeless people and indirectly improve the health of the general population.

HIV prevalence in the present study (3.8%) was lower than in a similar study in Tehran (6.4%) [7] and higher than in a study on Gypsy people (groups of people who do not take up permanent residence in any area and are homeless) in Shahr-E-Kord city, west of Iran (1.8%) [11]. In similar studies, HIV prevalence was reported at 4.2% in Mexico [9] and 8.5% in North America [12]. Based on a systematic review and meta-analysis study, HIV prevalence in the homeless population was estimated between 0.3 and 21.1% [3], which includes the HIV prevalence in the present study. HIV prevalence in the general population (blood donors) of Iran has been reported at 0.014% [13], [14], which in comparison with the results of this study indicates a high prevalence of infection in homeless people. In this study, it was demonstrated that the use of Kerack increased the risk of being HIV positive 7.1 times. As we found a high correlation between HIV and injecting drug use in this study (OR: 8.2, 95% CI: 2.9, 23.4), observed association between Kerack use and HIV can also be explained by the fact that Kerack, is a strong stimulant for injection [15]. Whereas some studies have shown that the use of crack (purified cocaine) increases the risk of HIV, as it increases risky sexual behaviours [16], [17], other studies have considered it to be an independent risk factor for HIV seroconversion among injection drug users [18], [19]. Examination of the different nature of Kerack (a derivative of heroin that is used in Iran [20]) and Crack (purified cocaine, which is used in other countries) could be helpful to identify the high-risk behaviours caused by them.

Although the prevalence of HCV in this study (23.3%) was lower than the prevalence in similar previous study carried out in Tehran in 2007 (42.8%) [7]; it was still however, in the estimated range of HCV infection in homeless people (3.9–36.2%) [3]. HCV prevalence in the general population of Iran has been reported at 0.065% [13], which in comparison with the results of this study, indicates a high risk of this infection for homeless people.

HBV prevalence in this study was 2.6%, which is lower than other similar studies in Iran: 34.7% in homeless people in Tehran [7] and 15.5% in the Gypsy peopleof Shahr-E-Kork city [11]. The prevalence of HBV in the general population (blood donors) of Iran has been reported at 0.8% (95% CI: 0.6, 0.9) [21], which is one third of the obtained HBV prevalence in this study and indicates that homeless people are a high risk group for HBV.

In this study, injecting drug use was a major risk factor for infection with HCV, HBV and HIV. Other studies on the homeless population have demonstrated that injecting drug use is associated with increased prevalence of HBV and HCV infection [22], [23]. In other studies on other groups, injecting drug use has been expressed as a factor that increases the risk of HCV, HBV and HIV [12], [24].

Today, the co-infection of HIV and viral hepatitis is a major health problem, with numerous social and economic consequences [25]. Co-infections of HIV/HCV and HIV/HBV in this study were reported at 2.9% and 0.5% respectively, which were lower than the reported prevalence in the previous study (6.4% and 5%, respectively). Triple viral co- infection in this study (0.5%) was 10 times lower than in the previous study (5%) [7].

It would seem that the obtained prevalence of viral infections is more substantial than in the previous study, as this study was carried out with a larger sample size and took into consideration all 5 receptor centres, and both men and women. However, lower levels of infections in this study in comparison to the previous study could be due to the development of health programmes and harm reduction programmes for these groups in recent years, which were implemented by the Municipality of Tehran.

Approximately 20% of women and 10% of men had a history of either selling sex or having sex with other men, respectively. Because of the illegality of such behaviours in Iran, it seems that information about this behaviour, as well as information relating to questions with stigma, is actually lower than in reality. Qualitative studies can help clarify the pattern of high-risk behaviours in this group.

As most of the homeless people in this study did not have appropriate employment, they may be involved in high-risk behaviours such as selling sex, using drugs, sharing needles and injecting with used syringes.

As a result of false positive results of the tuberculin skin test (TST) in vaccinated individuals and in those exposed to non-tuberculosis mycobacterium infections [26], the latent tuberculosis TST assessment has been replaced by whole-blood interferon-γ release assays (IGRAs) [27]. This study is the first to assess the prevalence of latent tuberculosis using IGRAs among homeless people in Iran. In this study the IGRA-positive prevalence was 46.71%, which is lower than the reported prevalence in similar studies in Italy (55.5%) [28], Mexico (50.7%) [9] and Japan (50.6%) [29].

The prevalence of active TB among homeless people is estimated to be 0.2–7.7% [3], which includes the obtained prevalence in the present study (1.22%). In similar studies, this prevalence is reported to be 0.39% in Mexico [9] and 1.52% in Japan [29]. In an antibiogram test, two persons (0.35%) were resistant to SM. Based on findings in the USA, there is a higher rate of TB among homeless people compared to non-homeless people, but they are not more drug resistant [30]. In this study, five persons were suspected as having active TB, but as there was no re-access to them, it was impossible to further evaluate them. Because of this limitation, there is an uncertainty about the prevalence of clinical TB in this study.

In the present survey, history of incarceration and its length of duration increased the risk of latent tuberculosis infection. This finding was also demonstrated in another study on prisoners in Pakistan (by TST) [31]. The current also showed that persons with a longer duration of homelessness had a higher risk of being positive for latent TB. It would appear that residing in closed areas, such as prisons and shelters, increases the risk of exposure to TB bacteria, and therefore increases the risk of infection [9].

HIV epidemics have led to the reactivation of latent tuberculosis and subsequently resulted in an increase of morbidity and mortality worldwide. In this study, the prevalence of HIV/IGRA+ (1.2%) and HIV/active TB (0.2%) were lower than the reported prevalence in a similar survey in Mexico (2.2% and 0.4% respectively) [9].

Although HIV prevalence in this study was not high, nevertheless, the high prevalence of HCV (23.3%), injection drug use (20.7%), and needle sharing (10.8%) can serve as a warning for future HIV epidemics among homeless people who are also intravenous drug users [32].

On the other hand, a considerably high rate of a history of selling sex in women (21.9%), men who had sex with other men (9.3%), sexual contacts outside of marriage (21.5%) and lack of condom use (66.7%) can induce HIV epidemics by sexual transmission in this group of people.

The rate of prevalence of all infections in this study was higher than that of the general population, which indicates that homeless people are at risk of these infections, and more attention to the health of these people is necessary.

The current study provided appropriate evidence of the health status and behaviours of homeless people in Tehran, i.e. patterns of drug use, sexuality, and viral and tuberculosis infections, which can be useful for health policy makers for future planning.

Although treatment of TB patients and HIV infected cases is free of charge, the treatment of viral hepatitis is very expensive for homeless people, as they do not have health insurance. So decision-making with respect to the treatment of HBV and HCV patients seems to be an important issue.

It is recommended that the knowledge of homeless people regarding prevention methods of these diseases is increased, to reduce their transmission rate. Our findings highlighted that more attention to the health of homeless people is seriously needed. TB and other infections were relatively common among this group. However, it seems that some predictors such as the history of drug use, imprisonment, and even age, may be used to efficiently find more vulnerable individuals.
